# Dengue Hemorrhagic Fever Complicated by Complete Atrioventricular Block: A Case Report

**DOI:** 10.7759/cureus.84611

**Published:** 2025-05-22

**Authors:** Tanad Abshir, Ali Al Hassani, Zaid Al Hassani, Aqeel Saleem

**Affiliations:** 1 Internal Medicine, Sheikh Tahnoon Bin Mohammed Medical City, Al Ain, ARE; 2 Infectious Disease, Sheikh Tahnoon Bin Mohammed Medical City, Al Ain, ARE; 3 College of Medicine, University of Sharjah, Sharjah, ARE

**Keywords:** complete heart block, dengue fever, dengue hemorrhagic fever, dengue shock syndrome, heart block

## Abstract

Dengue virus (DENV) infection is a common mosquito-borne disease with a broad clinical spectrum ranging from mild febrile illness to severe manifestations such as dengue haemorrhagic fever (DHF) and dengue shock syndrome. While hematologic and vascular complications are well-documented, cardiac involvement, such as conduction abnormalities, is less well-known but potentially fatal. A 53-year-old male with a five-day history of fever, fatigue, and dizziness presented in hemodynamic instability with severe bradycardia and hypotension. An ECG showed complete heart block, requiring the urgent insertion of a temporary pacemaker. Transthoracic echocardiogram demonstrated mildly reduced left ventricular systolic function (left ventricular ejection fraction = 45%). Laboratory investigations revealed thrombocytopenia with a platelet count of 109 × 10⁹/L. The patient had no known allergic conditions. Based on the clinical presentation and high index of suspicion, a dengue test was performed, confirming the infection through positive IgM serology and reverse transcriptase-polymerase chain reaction, which identified the DENV-2 serotype. Despite supportive care, the patient did not recover a normal cardiac rhythm and ultimately required a permanent pacemaker. This case highlights the potential for complete heart block as a severe complication of DHF.

## Introduction

Dengue virus (DENV) infection is among the most prevalent mosquito-borne diseases, affecting millions annually in tropical and subtropical regions [1]. It is transmitted through the bite of female *Aedes aegypti* or *Aedes albopictus* mosquitoes carrying one or more of the four DENV serotypes, namely, DENV-1, DENV-2, DENV-3, or DENV-4 [2]. While primary infections are often asymptomatic or present with mild febrile illness, severe cases can lead to complications such as coagulopathy, increased vascular permeability, and vascular fragility, hallmarks of dengue hemorrhagic fever (DHF). If left unmanaged, DHF can progress to dengue shock syndrome (DSS), a life-threatening condition with high mortality risk [3].

Cardiac involvement in dengue is multifactorial, resulting from direct viral invasion of myocardial tissue, immune-mediated injury, and systemic inflammation [4]. The virus can infect cardiomyocytes and disrupt conduction, while elevated cytokines in severe dengue contribute to myocardial inflammation and functional impairment [4].

This case report presents a rare occurrence of complete heart block as a complication of DHF and examines the broader implications of cardiac involvement in dengue infections. Recognizing and understanding these complications are crucial for improving patient outcomes, reducing morbidity, and preventing mortality.

## Case presentation

**Figure 1:**
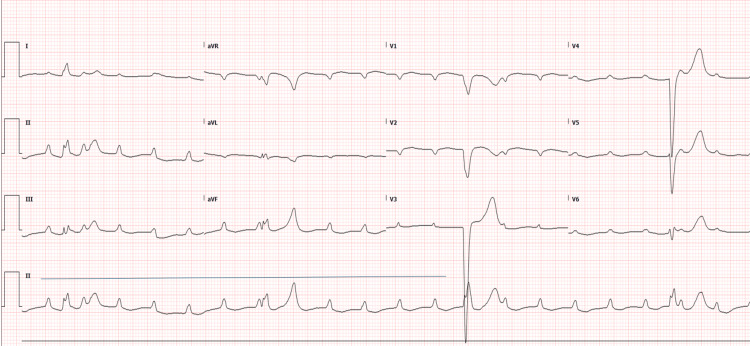
ECG showing complete atrioventricular dissociation, obtained during the initial emergency department assessment.

**Figure 2:**
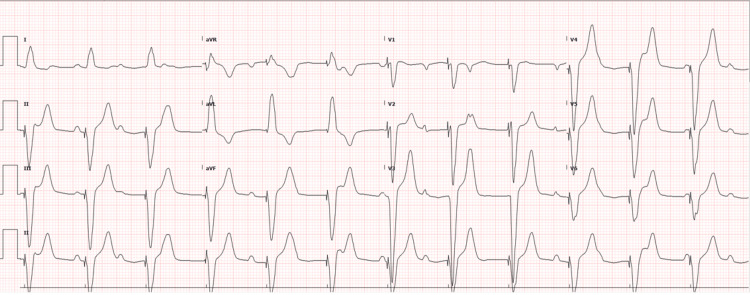
Repeat ECG following temporary pacemaker insertion demonstrating paced rhythm.

A 53-year-old male cattle farm worker with a history of type 2 diabetes mellitus and hypertension presented to the emergency department on day one with a five-day history of fever, fatigue, chills, dizziness, and poor oral intake. On arrival, he was agitated, confused, and unable to follow commands. There was no recent travel or sick contact. Initial evaluation revealed hemodynamic instability with severe bradycardia (heart rate = 38 beats/minute) and hypotension (blood pressure = 75/35 mmHg). Systemic examination was otherwise unremarkable, with no signs of hemorrhage, ascites, or pleural effusion. The admission ECG showed complete heart block (Figure 1). Due to his critical condition, the patient underwent urgent temporary pacemaker insertion within the first hour of arrival, achieving a paced rhythm at 80 beats/minute (Figure 2).

Initial laboratory investigations (Table 1) revealed thrombocytopenia (109 × 10⁹/L), elevated hemoglobin (178 g/L), suggestive of hemoconcentration, and mildly elevated troponin T (62.7 ng/L) and N-terminal pro-B-type natriuretic peptide (288 ng/L). These findings, alongside hypotension, met World Health Organization (WHO) criteria for DHF due to thrombocytopenia, plasma leakage (hemoconcentration), and hemodynamic compromise [5]. Inflammatory markers (C-reactive protein, fibrinogen) were within the normal range. Hepatitis and HIV serologies were negative.

**Table 1:** Laboratory findings on admission.

Laboratory parameter	On admission	Normal value
Sodium	133 mmol/L	135–145 mmol/L
Potassium	4.6 mmol/L	3.5–4.5 mmol/L
Chloride	103 mmol/L	98–107 mmol/L
Magnesium	0.66 mmol/L	0.66–1.07 mmol/L
Phosphate	0.90 mmol/L	0.81–1.45 mmol/L
Calcium	2.22 mmol/L	2.10–2.60 mmol/L
Calcium corrected level	2.46 mmol/L	2.10–2.60 mmol/L
Creatinine	68 mmol/L	62–106 mmol/L
Urea	9.20 mmol/L	2.80–8.10 mmol/L
White blood cell count	8.7 × 10^9^/L	4.5–11.0 × 10^9^/L
Red blood cell count	6.21 × 10^12^/L	4.20–5.60 × 10^12^/L
Hemoglobin	178 g/L	131–172 g/L
Platelets	109 × 10^9^/L	140–400 × 10^9^/L
Lactic acid	1.7 mmol/L	0.5–2.2 mmol/L
Procalcitonin	0.73 ng/mL	≤0.50 ng/mL
C-reactive protein	9.2 mg/L	≤5.0 mg/L
troponin	62.7 ng/L	≤14.0 ng/L
N-terminal pro-B-type natriuretic peptide	288 ng/L	0.0–121.0 ng/L
Prothrombin time	10.7 seconds	9.5–12.5 seconds
Activated partial thromboplastin time	30.2 seconds	22.2–34.2 seconds
International normalized ratio	0.99	0.87–1.15
Fibrinogen level	3.16 g/L	1.50–3.87 g/L
HIV	Negative	Negative
Hepatitis Bs antigen	Negative	Negative
Hepatitis Bs antibody	0.03 mIU/L	>10 mIU/L
Hepatitis B core antibody	Negative	Negative
Hepatitis C antibody	Negative	Negative
Hepatitis A IgG	Positive	Negative
Hepatitis A IgM	Negative	Negative

Given the patient’s presentation with a five-day history of fever, fatigue, dizziness, and hemodynamic instability, a dengue test was conducted due to a high index of suspicion. Dengue IgM serology and reverse transcriptase-polymerase chain reaction confirmed DENV-2 infection by day two. In response, the patient was initiated on intravenous antipyretics and fluid therapy to manage symptoms and prevent complications.

**Figure 3:**
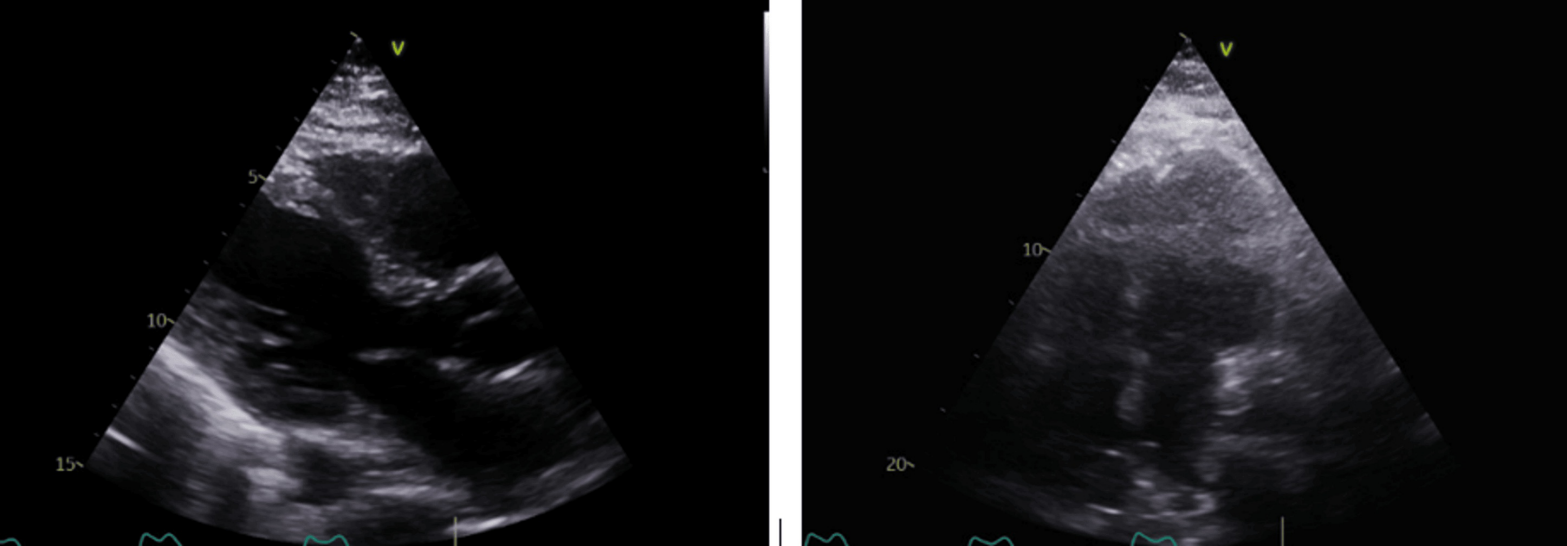
Transthoracic echocardiogram (showing mildly reduced left ventricular systolic function (left ventricular ejection fraction = 45%).

Simultaneously, a transthoracic echocardiogram was performed on day two post-admission (Figure 3), revealing a normal left ventricular (LV) size with mild LV systolic dysfunction and an LV ejection fraction of 40-45%. The interventricular septum appeared dysynchronous due to temporary pacing. No regional wall motion abnormalities were observed. Additionally, there was mild tricuspid regurgitation, normal right ventricular size and function, and no significant pulmonary hypertension.

While dengue-related myocarditis was suspected given elevated biomarkers, cardiac MRI and biopsy were deferred due to clinical instability. There was no evidence of myocardial ischemia, as the patient had no chest pain, serial troponin levels showed only a mild elevation without dynamic changes, and there were no ischemic changes on ECG. Drug toxicity was also ruled out, as there was no history of exposure to atrioventricular node-blocking agents. Additionally, electrolyte imbalance was excluded, with normal potassium (4.6 mmol/L) and magnesium (0.66 mmol/L) levels. The temporary pacemaker lead was visualized in the right ventricle.

Despite seven days of supportive care and temporary pacing, the patient did not regain atrioventricular node function. Given the persistent complete heart block and absence of rhythm recovery, a permanent pacemaker was implanted on day eight per established 2021 European Society of Cardiology Guidelines for high-degree atrioventricular block without reversible cause [6].

The patient tolerated the procedure well and was discharged in stable condition on day 10. Outpatient cardiology follow-up, including Holter monitoring over three months, confirmed the absence of intrinsic rhythm recovery. These follow-ups also aimed to monitor pacemaker function and assess for any late recovery of atrioventricular conduction. No spontaneous atrioventricular conduction was observed during hospitalization or follow-up, supporting the diagnosis of irreversible conduction system injury.

## Discussion

DENV infection remains a significant global health concern, particularly in tropical and subtropical regions, with millions of cases reported annually [1]. The virus, belonging to the Flaviviridae family, is primarily transmitted by *Aedes aegypti *and, in some instances, *Aedes albopictus* mosquitoes [2]. Among the four DENV serotypes, DENV-2 and DENV-3 have been more frequently associated with severe manifestations, including cardiac complications [1,3].

Epidemiological estimates suggest that approximately 390 million dengue infections occur worldwide each year, with around 96 million cases exhibiting clinical symptoms ranging from mild to severe [7]. Additionally, nearly 4 billion people are considered at risk, with a significant portion living in urban and peri-urban areas [7]. Over the past two decades, the number of dengue cases reported to the WHO has surged dramatically, increasing eightfold from 505,430 cases in 2000 to 5.2 million in 2019 [7].

The clinical spectrum of dengue varies from mild febrile illness to life-threatening conditions such as DHF and DSS [3]. According to WHO guidelines, dengue is classified based on severity into the following three categories: dengue without warning signs, dengue with warning signs, and severe dengue [8]. Severe cases often present with plasma leakage, hemorrhagic manifestations, and multi-organ dysfunction, which contribute to morbidity and mortality [8].

The pathophysiology of cardiac involvement is multifactorial, involving direct viral invasion of myocardial tissue, immune-mediated damage, and systemic inflammation. DENV has been shown to infect cardiomyocytes and cardiac endothelial cells, triggering apoptosis and disrupting electrical conduction pathways [4]. Additionally, the cytokine storm characteristic of severe dengue, marked by elevated interleukin 6, tumor necrosis factor-alpha, and NS1 antigen levels, induces myocardial inflammation, microvascular leakage, and interstitial edema, further compromising cardiac function [4]. The complete heart block in this patient, despite normal inflammatory markers, likely resulted from direct DENV invasion of the conduction system or immune-mediated injury, as histopathological studies in dengue-associated cardiac complications demonstrate myocardial necrosis and lymphocytic infiltration [4].

Although dengue is primarily known for its hematologic and vascular complications, cardiac involvement is increasingly recognized, particularly in association with DENV-2 and DENV-3 [8]. Cardiac manifestations may result from direct viral invasion of myocardial tissue, immune-mediated injury, or autonomic dysfunction [9]. Reported cardiac manifestations include T-wave abnormalities, ST-segment changes, sinus pauses, atrial and ventricular ectopics, bundle branch blocks, and varying degrees of heart block [9]. While bradycardia is more frequently observed during the defervescence and convalescent phases, complete atrioventricular dissociation remains a rare but serious complication [9]. Cardiac complications have been documented in 12.5% of dengue patients in an Indian study (n = 120), 62.5% in a Sri Lankan cohort (n = 120), and 35% in a study conducted in Vietnam [10-12].

In dengue-related atrioventricular block, temporary pacing is indicated for hemodynamic instability (e.g., syncope, hypotension, or heart failure), while permanent pacemaker implantation (PPI) is typically deferred for two to six weeks to assess reversibility, as most conduction abnormalities resolve spontaneously during recovery [5,13]. Prognosis is generally favorable, with studies reporting resolution of atrioventricular block in 70-80% of cases within four weeks [12,14]. However, PPI may be warranted if conduction defects persist beyond six to eight weeks or if myocardial necrosis (e.g., elevated troponin, MRI evidence) suggests irreversible damage [12,13]. Debate exists due to limited long-term data, with some experts advocating cautious observation given dengue’s transient nature, while others prioritizing PPI in cases with persistent symptomatic bradycardia or high-grade block [13,14].

Recognizing and understanding these cardiac complications in dengue is crucial, as early diagnosis and appropriate management can significantly reduce morbidity and mortality [9]. This case highlights the importance of cardiac monitoring in dengue with cardiac complication cases, particularly in patients presenting with conduction abnormalities [1,9].

In our study, our patient was diagnosed with dengue fever complicated by complete heart block, initially requiring urgent intervention with a temporary pacemaker due to hemodynamic instability [1,3,9]. Despite supportive care, he did not return to normal cardiac rhythm, leading to the decision for PPI [9].

## Conclusions

DENV infection, particularly severe dengue with cardiac complications, can lead to life-threatening conduction abnormalities such as complete heart block, as exemplified by this patient who ultimately required PPI due to irreversible atrioventricular nodal dysfunction. While transient bradyarrhythmias are common in dengue, complete heart block, though rare, demands targeted cardiac monitoring in high-risk patients with bradycardia, syncope, or hypotension to enable timely intervention. Routine ECG or telemetry in such cases may improve early detection of conduction defects, guided by symptom-driven risk stratification to avoid over-testing. Although dengue-related complete heart block often resolves spontaneously, persistent block beyond six to eight weeks or evidence of myocardial necrosis warrants permanent pacing, as seen here. Long-term follow-up is advised to assess recovery in transient cases and device function in irreversible ones, emphasizing the need for individualized management in this evolving clinical landscape. However, the generalizability of these findings is limited by the single-case nature of this report, underscoring the necessity for larger studies to define risk factors, optimal pacing strategies, and long-term outcomes in dengue-associated complete heart block.
